# Safety of 48 months of elagolix with add-back therapy for endometriosis-associated pain

**DOI:** 10.1016/j.xagr.2025.100584

**Published:** 2025-11-07

**Authors:** Jin Hee Kim, Charles E. Miller, James A. Simon, James W. Thomas, Anna Chan, Michael C. Snabes

**Affiliations:** 1 Department of Obstetrics and Gynecology, Columbia University, New York, NY (Kim); 2 Department of Minimally Invasive Gynecologic Surgery, Advocate Lutheran General Hospital, Park Ridge, IL (Miller); 3 The Advanced Gynecologic Surgery Institute, Naperville, IL (Miller); 4 George Washington University School of Medicine, IntimMedicine Specialists, Washington, DC (Simon); 5 AbbVie Inc., North Chicago, IL (Thomas, Chan and Snabes)

**Keywords:** bone mineral density, elagolix, endometriosis, estradiol, pain, pelvic pain, safety

## Abstract

**Background:**

Elagolix (ELA) 200 mg twice daily is an oral treatment approved for moderate-to-severe endometriosis-associated pain. However, the clinical use of ELA is limited by potential hypoestrogenic effects, including the loss of bone mineral density (BMD). The addition of hormonal add-back (AB) therapy (1-mg estradiol/0.5-mg norethindrone acetate once daily) may attenuate BMD loss.

**Objectives:**

To provide long-term safety results from a 48-month phase 3 trial assessing ELA+AB therapy in premenopausal women with moderate-to-severe endometriosis-associated pain.

**Study Design:**

This multicenter, phase 3 trial was conducted between July 7, 2017, and December 6, 2023, and included a 12-month double-blind treatment period, a 36-month open-label extension period, and a 12-month post-treatment follow-up (PTFU) period. At the beginning of the double-blind period, premenopausal women (aged 18−49 years) with moderate-to-severe endometriosis-associated pain were randomized 4:1:2 to ELA+AB therapy, ELA monotherapy for 6 months followed by ELA+AB therapy for the remaining 6 months, or placebo. After the double-blind treatment period, all patients were treated with open-label ELA+AB therapy (referred to as the ELA+AB/ELA+AB, ELA/ELA+AB, and placebo/ELA+AB groups) for the remainder of the 36-month open-label treatment period. Safety was assessed throughout the open-label treatment period. BMD was measured by dual-energy X-ray absorptiometry in the lumbar spine, total hip, and femoral neck during the screening period and every 6 months throughout the study. BMD was analyzed using an analysis of covariance model with treatment as the main effect and baseline values as covariates.

**Results:**

Among 380 patients who entered the open-label period, 140 (36.8%) completed the treatment period, and 240 (63.2%) discontinued, primarily due to withdrawn consent and adverse events (AEs). In total, 159 (74.0%) patients in the ELA+AB/ELA+AB group, 41 (69.5%) patients in the ELA/ELA+AB group, and 80 (75.5%) patients in the placebo/ELA+AB group reported at least 1 treatment-emergent adverse event (TEAE) during the open-label period. TEAEs were generally mild or moderate and nonserious. The most commonly reported TEAEs were COVID-19, bone density decreases, and sinusitis. No serious AEs were reported in >1 patient in any study group. BMD remained generally stable, with a least squares mean percent decrease from baseline to week 48 <2% in all groups and at all anatomical sites. Similarly, the least squares mean decrease from baseline in BMD was <2% in the ELA+AB/ELA+AB group at all anatomical sites and all time points throughout the study period. For patients in the ELA+AB/ELA+AB group with ≥48 months of treatment and reduced BMD at the final on-treatment visit, the majority (lumbar spine, 13 [59.1%]; total hip, 8 [53.3%]; femoral neck, 12 [57.1%]) partially or fully recovered at month 12 of the PTFU period.

**Conclusions:**

This study extends findings from a previously reported 12-month analysis of the efficacy and safety of ELA+AB therapy in premenopausal women with moderate-to-severe endometriosis-associated pain. The safety profile of ELA+AB therapy was generally consistent with that observed in prior studies. BMD remained mostly stable through 48 months of continuous treatment. This analysis represents the longest safety study of ELA+AB therapy to date and supports long-term treatment with ELA+AB therapy.

**AJOG Global Reports at a Glance**

**Why was this study conducted?**

This study evaluated the safety of elagolix with hormonal add-back (ELA+AB) therapy for up to 48 months in premenopausal women with endometriosis-associated pain in a phase 3 trial.

**Key findings?**

ELA+AB therapy demonstrated no new tolerability concerns. Bone mineral density (BMD) generally remained stable (<2% decrease from baseline) throughout treatment. Patients treated with ELA+AB therapy who experienced decreases from baseline in BMD typically showed at least partial recovery after treatment cessation.

**What does this study add to what is known?**

This study is the longest safety assessment of ELA+AB therapy in premenopausal women with moderate-to-severe endometriosis-associated pain. These results are generally consistent with prior studies of ELA+AB, indicating ELA+AB therapy may provide a long-term treatment option for women with endometriosis-associated pain.

## Introduction

Endometriosis, an inflammatory condition characterized by aberrant growth of endometrial tissue,^1^^,^^2^ affects ≈10% of women of childbearing age, and can be associated with retrograde menstruation, fibrosis, adhesions, anatomical distortion, and infertility.^3^^,^^4^ Symptoms of endometriosis, including dysmenorrhea (DYS) and nonmenstrual pelvic pain (NMPP), can be disabling, substantially impacting patients’ physical and mental quality of life.^1^^,^^5^ Endometriosis pathophysiology relies on estrogen for proliferation and extrauterine implantation of endometrial tissue.^6^^,^^7^

As a chronic condition, endometriosis requires long-term management.^6^ Available therapies aim to decrease symptoms and improve quality of life.^2^ Treatments include surgery and medications (e.g., nonsteroidal anti-inflammatory drugs [NSAIDs], progestin-based therapies, gonadotropin-releasing hormone [GnRH] analogues).^2^ However, surgery is invasive, and repeat procedures can be required within 5 years. Efficacy of progestin therapies may be limited by progestin resistance or intolerable side effects, and long-term use of GnRH agonists and antagonists is limited by potential hypoestrogenic effects, including hot flushes, hyperlipidemia, and loss of bone mineral density (BMD).^8^, ^9^, ^10^ Nonsurgical therapies that safely provide long-term symptom relief to women with endometriosis are needed.

Elagolix (ELA) is an oral, nonpeptide GnRH antagonist approved for treating endometriosis-associated pain.^11^ Despite efficacy for treating DYS and NMPP in initial phase 3 studies,^8^^,^^12^ ELA 200 mg once daily is limited to use up to 6 months due to observed hypoestrogenic effects, including bone loss.^8^^,^^12^^,^^13^ After 12 months of ELA 200-mg twice-daily treatment, clinically meaningful reductions from baseline in DYS (76–78% of patients) and NMPP (67–69%) were reported; however, BMD was also reduced to −3.6% to −3.9% in the lumbar spine.^12^ The addition of hormonal add-back (AB) therapy (1-mg estradiol/0.5-mg norethindrone acetate once daily) attenuates the effects of ELA monotherapy on BMD loss while maintaining efficacy for endometriosis-associated pain.^8^^,^^13^ BMD loss was more pronounced after 6 months of 200-mg twice-daily ELA monotherapy (lumbar spine, −2.9%; total hip, −1.7%; femoral neck, −1.9%) than after 12 months of ELA+AB therapy (lumbar spine, −1.1%; total hip, −0.9%; femoral neck, −0.5%);^13^ 64% and 54% of patients experienced meaningful improvements in DYS and NMPP (both *P* <0.01 vs. placebo), respectively.^13^

Data regarding long-term safety of ELA+AB therapy are limited. This analysis builds on previously published results from a 12-month, double-blind phase 3 trial assessing safety and efficacy of ELA+AB therapy for moderate-to-severe endometriosis-associated pain.^13^ We report safety results during the 36-month open-label extension period, which included cumulative treatment with ELA+AB therapy to 48 months, and 12-month post-treatment follow-up (PTFU) period.

## Materials and methods

### Study design and participants

Detailed methods for this study have been published previously.^13^ Briefly, this 48-month, phase 3 trial assessed efficacy and safety of ELA+AB therapy for endometriosis-associated pain. The study was conducted between July 7, 2017, and December 6, 2023, at 197 sites across Canada, Puerto Rico, and the United States, and included a 12-month double-blind treatment period, a 36-month open-label treatment period, and a 12-month PTFU period. During the initial 12-month period, patients were randomized 4:1:2 using interactive response technology (IRT) to receive oral ELA+AB therapy, 200 mg ELA twice daily (first 6 months) followed by ELA+AB therapy (remaining 6 months), or placebo. At the end of the 12-month placebo-controlled treatment period, all patients received open-label ELA+AB therapy until study month 48, regardless of initial double-blind treatment (treatment groups are denoted here as the ELA+AB/ELA+AB, ELA/ELA+AB, and placebo/ELA+AB treatment groups, respectively). The PTFU period lasted up to 12 months after the last dose of study drug, regardless of whether a patient completed study drug treatment or discontinued prematurely.

This study included premenopausal women aged 18−49 years with a surgically confirmed diagnosis of endometriosis within the last 10 years and moderate-to-severe endometriosis-associated pain (≥2 days of moderate or severe DYS and ≥2 days of moderate or severe NMPP with an average NMPP score ≥1.0 or ≥4 days of moderate or severe NMPP with an average NMPP score ≥0.5) within 35 days before study day 1. Patients were excluded if they had nonendometriosis chronic pelvic pain (e.g., interstitial cystitis, adenomyosis [dominant condition diagnosed by magnetic resonance imaging or ultrasound], fibroids, pelvic inflammatory disease, nonendometriosis-related pelvic adhesive disease, or irritable bowel syndrome) or other chronic pain requiring long-term analgesic therapy and which the investigator determined could interfere with the assessment of endometriosis-related pain, used systemic corticosteroids for >14 days within 3 months of screening, had a history of major depression or post-traumatic stress disorder within 2 years before screening or any other major psychiatric disorder at any time, had a history of attempted suicide within 1 year before screening or study day 1, had osteoporosis or other metabolic bone disease, had any condition that could interfere with adequate dual energy X-ray absorptiometry (DXA) measurements, had screening DXA measurements ≥2.0 standard deviations below normal, or had any conditions contraindicated for AB therapy.

The study was conducted in accordance with Good Clinical Practice, the International Council for Harmonization, the Declaration of Helsinki, and all applicable local regulations and guidelines. All relevant study materials (including protocols, informed consent forms, etc) received independent ethics committee/institutional review board (IRB) approval before the trial (approval received March 4, 2016; IRB reference number: 201601247). Patients provided written informed consent before participation.

### Assessments

Primary and key secondary efficacy endpoints for the 12-month double-blind treatment period have been reported.^13^

This analysis focused on long-term safety of ELA+AB therapy during the 36-month open-label treatment period. Safety assessments included treatment-emergent adverse events (TEAEs) and the change from baseline (CFB) in BMD. TEAEs were coded using the Medical Dictionary for Regulatory Activities v26.1 and were defined as any TEAE starting on or after the first day of treatment until 30 days after the last dose of study drug. TEAEs beginning in the placebo-controlled treatment period and extending into the open-label treatment period were not included in the safety results for the open-label period. Serious AEs (SAEs) were defined as any TEAEs that resulted in death or new or prolonged hospitalization, were considered life-threatening, were associated with congenital anomaly or fetal loss, resulted in persistent or significant disability, or required medical/surgical intervention. TEAEs were assessed based on their incidence, potential relationship with the study drug, possible association with study discontinuation, severity (mild [transient/easily tolerated], moderate [causes discomfort or interrupts activities], or severe [considerable interference with activities, may be incapacitating or life-threatening]), and seriousness.

BMD was assessed at the lumbar spine, total hip, and femoral neck via DXA during screening and every 6 months throughout the treatment and PTFU periods. DXA scanning was performed using either the Lunar iDXA (GE HealthCare Lunar, Madison, WI, USA) or the Horizon® DXA System (Hologic, Inc, Marlborough, MA, USA), with interpretations performed by a blinded central reader. Patients were required to discontinue treatment if they had a Z-score (patients aged <40 years at screening) or a T-score (patients aged ≥40 years) less than −2.5, or if they experienced a decrease in BMD from baseline >8% in any anatomic area. BMD decreases resulting in discontinuation were documented as adverse events.

### Statistical analysis

As this was a descriptive analysis, enrollment in the open-label extension was at the discretion of the investigators, and this study was not powered to detect a safety-specific outcome. Demographics and baseline characteristics were analyzed in the full analysis set (randomized patients who received ≥1 dose of study drug). Safety analyses were performed using the safety analysis set (patients who received ≥1 dose of study drug). Data were analyzed according to the treatment received. Categorical variables were reported as the number and percentage of patients, and continuous variables were summarized using descriptive statistics. Safety analyses were based on observed data with no imputation for missing data. Exposure-adjusted adverse event rates were calculated based on the number of events per 100 patient-years.

Percent CFB in BMD was assessed using an analysis of covariance model that included treatment as a main effect and baseline values of the corresponding parameters as covariates. At each time point, patients were excluded from the analysis if their BMD was measured using a different densitometer from that used for their screening visit scan. BMD recovery during the PTFU period among patients who experienced a decrease in BMD at their final on-treatment visit was calculated as follows:

$$\frac{\left(\%\text{CFB}\;\text{at}\;\text{final}\;\text{on}-\text{treatment}\;\text{visit}-\%\text{CFB}\;\text{at}\;\text{each}\;\text{PTFU}\;\text{visit}\right)}{\left(\%\text{CFB}\;\text{at}\;\text{final}\;\text{on}-\text{treatment}\;\text{visit}\right)}$$

Results for percent recovery include only those patients with available data at treatment month 48 and PTFU month 12.

## Results

### Patients

**Figure 1:**
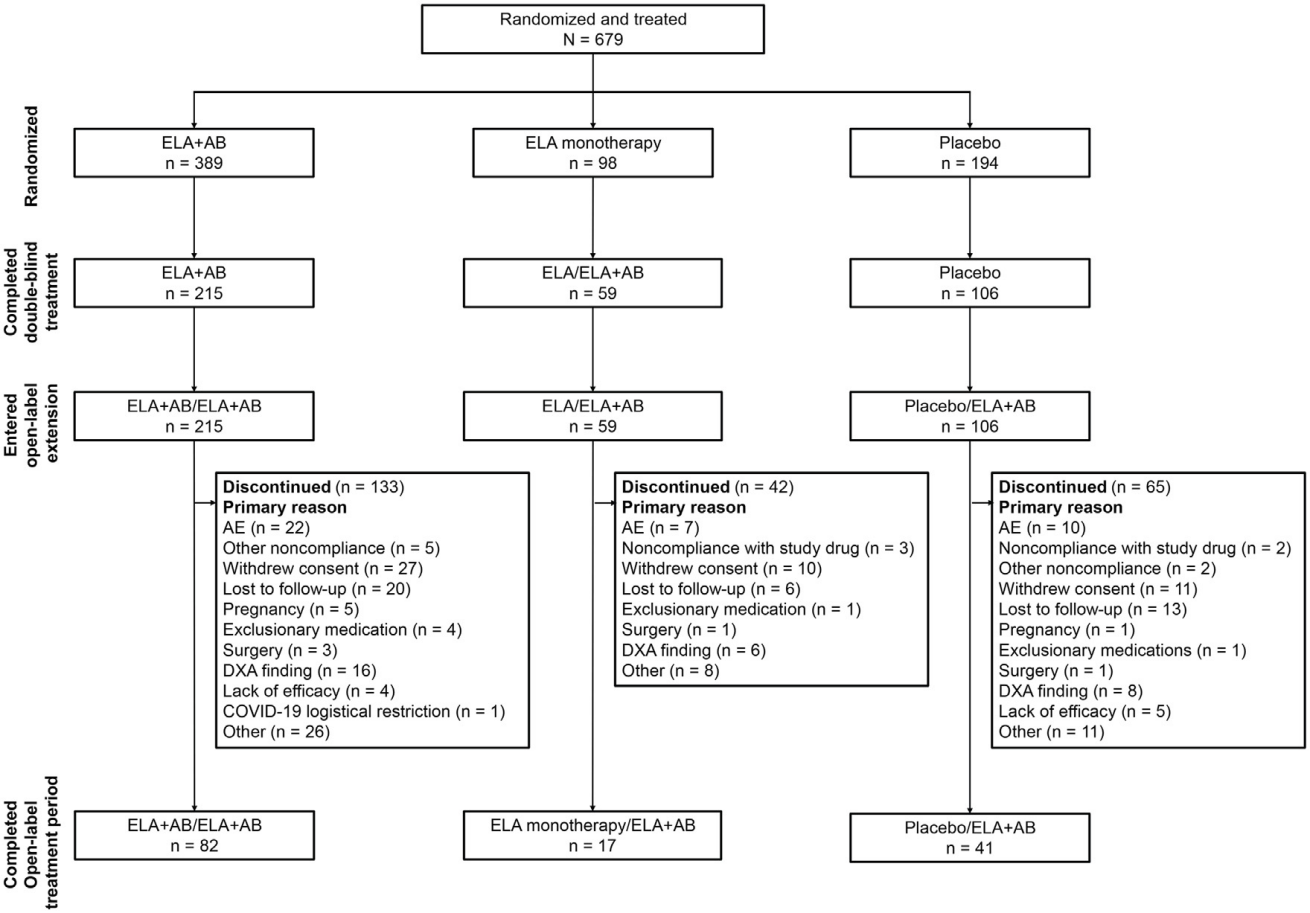
Patient disposition AB, hormonal add-back therapy (estradiol 1 mg/norethindrone acetate 0.5 mg once daily); AE, adverse event; DXA, dual-energy X-ray absorptiometry; ELA, elagolix 200 mg twice daily. Patient disposition for the double-blind treatment period, including reasons for discontinuation, have been previously published.^13^

Baseline demographics and patient disposition for the double-blind treatment period have been published.^13^ At baseline of the double-blind treatment period, demographics and clinical characteristics were generally balanced between groups (Table 1). Patients had a mean (SD) age of 32.3 (6.7) years in the ELA+AB/ELA+AB group, 32.5 (6.4) years in the ELA/ELA+AB group, and 33.1 (6.7) years in the placebo/ELA+AB group. Most patients were White (ELA+AB/ELA+AB, 84.3%; ELA/ELA+AB, 76.3%; placebo/ELA+AB, 82.4%). Of the 380 patients who entered the open-label ELA+AB treatment period, 140 (36.8%) completed the treatment period and 240 (63.2%) discontinued. The most common primary reasons for discontinuation were withdrawn consent (n=48 [20.0%]), “other” (n=45 [18.8%]), TEAE (n=39 [16.3%]), and lost to follow-up (n=39 [16.3%]; Figure 1). T- or Z-score less than −2.5 or BMD decrease >8% was the primary reason for discontinuation for 30 (12.5%) patients. During the open-label treatment period, patients had a mean (SD) exposure to ELA+AB therapy of 623.4 (365.5) days, with similar exposure to study drug across treatment groups (Supplemental Table 1).

**Table 1:** Baseline demographics

Characteristic	ELA+AB/ELA+AB (n=389)	ELA/ELA+AB (n=97)	Placebo/ELA+AB (n=193)
Age, years, mean (SD)	32.3 (6.7)	32.5 (6.4)	33.1 (6.7)
Female, n (%)	389 (100)	97 (100)	193 (100)
BMI, kg/m^2^, mean (SD)	29.4 (7.3)^a^	29.5 (7.4)	29.2 (6.8)^b^
Race, n (%)			
White	328 (84.3)	74 (76.3)	159 (82.4)
Black	49 (12.6)	19 (19.6)	31 (16.1)
Asian	4 (1.0)	0	0
American Indian or Alaska Native	1 (0.3)	0	1 (0.5)
Native Hawaiian or other Pacific Islander	1 (0.3)	0	0
Multiple	6 (1.5)	4 (4.1)	2 (1.0)
Ethnicity, n (%)			
Hispanic or Latino	58 (14.9)	11 (11.3)	37 (19.2)
Not Hispanic or Latino	331 (85.1)	86 (88.7)	156 (80.8)
Time since endometriosis diagnosis, months, mean (SD)	47.2 (32.4)^c^	44.1 (32.2)	50.1 (32.8)
Method of endometriosis diagnosis, n (%)			
Laparoscopy	360 (95.7)^d^	91 (94.8)^e^	181 (94.8)^f^
Laparotomy	16 (4.3)^d^	5 (5.2)^e^	10 (5.2)^f^

AB, hormonal add-back therapy (estradiol 1 mg/norethindrone acetate 0.5 mg once daily); BMI, body mass index; ELA, elagolix 200 mg twice daily.

Baseline demographics and clinical characteristics have been published previously.^13^

a n=386.

b n=191.

c n=388.

d n=13 patients missing data.

e n=1 patient missing data.

f n=1 patient missing data.

Kim. Safety of 48 months of elagolix with add-back therapy. Am J Obstet Gynecol 2025.

### Long-term safety

During the 36-month open-label treatment period, 159 (74.0%) patients in the ELA+AB/ELA+AB group, 41 (69.5%) patients in the ELA/ELA+AB group, and 80 (75.5%) patients in the placebo/ELA+AB group reported at least 1 TEAE (Table 2). Rates of SAEs and severe TEAEs were generally low (<11% in any group), and most TEAEs did not result in study drug discontinuation. No deaths occurred during the study. Similar results were observed when examining exposure-adjusted event rates (Supplemental Table 2).

**Table 2:** Overview of TEAEs during the open-label treatment period

TEAEs, n (%)^a^^,^^b^	ELA+AB/ELA+AB (n=215)	ELA/ELA+AB (n=59)	Placebo/ELA+AB (n=106)
Any TEAE	159 (74.0)	41 (69.5)	80 (75.5)
TEAE possibly related to study drug^c^	80 (37.2)	22 (37.3)	52 (49.1)
Severe TEAE	23 (10.7)	3 (5.1)	11 (10.4)
SAE	10 (4.7)	2 (3.4)	7 (6.6)
TEAE leading to discontinuation	39 (18.1)	11 (18.6)	19 (17.9)
Deaths	0	0	0

AB, hormonal add-back therapy (estradiol 1 mg/norethindrone acetate 0.5 mg once daily); ELA, elagolix 200 mg twice daily; SAE, serious adverse event; TEAE, treatment-emergent adverse event.

a Includes all patients who received ≥1 dose of ELA+AB therapy.

b Patients are counted once in each row, regardless of the number of events they may have reported.

c As assessed by the investigator.

The most common TEAEs during the open-label treatment period were COVID-19 (ELA+AB/ELA+AB, 30 [14.0%]; ELA/ELA+AB, 7 [11.9%]; placebo/ELA+AB, 9 [8.5%]), BMD decreased (ELA+AB/ELA+AB, 21 [9.8%]; ELA/ELA+AB, 6 [10.2%]; placebo/ELA+AB, 7 [6.6%]), and sinusitis (ELA+AB/ELA+AB, 18 [8.4%]; ELA/ELA+AB, 5 [8.5%]; placebo/ELA+AB, 9 [8.5%]; Table 3). Most TEAEs reported in the open-label treatment period were nonserious, with no more than 1 patient in any treatment group reporting any particular SAE (Table 4). While rates of severe TEAEs were low overall, the most common severe TEAEs generally aligned with known effects of endometriosis and hormonal therapy, including night sweats and mood changes (Supplemental Table 3). Among the most common TEAEs considered by investigators to be related to the study drug were BMD decrease, depression, hot flush, weight increased, and night sweats (Supplemental Table 4).

**Table 3:** Most common TEAEs during the open-label treatment period

TEAEs Occurring in ≥5.0% of the ELA+AB/ELA+AB Group, n (%)^a^^,^^b^	ELA+AB/ELA+AB (n=215)	ELA/ELA+AB (n=59)	Placebo/ELA+AB (n=106)
COVID-19	30 (14.0)	7 (11.9)	9 (8.5)
Bone density decreased	21 (9.8)	6 (10.2)	7 (6.6)
Sinusitis	18 (8.4)	5 (8.5)	9 (8.5)
Anxiety	17 (7.9)	3 (5.1)	7 (6.6)
Urinary tract infection	16 (7.4)	5 (8.5)	6 (5.7)
Back pain	15 (7.0)	2 (3.4)	2 (1.9)
Depression	15 (7.0)	2 (3.4)	2 (1.9)
Vulvovaginal mycotic infection	14 (6.5)	4 (6.8)	3 (2.8)
Upper respiratory tract infection	13 (6.0)	1 (1.7)	6 (5.7)
Nausea	12 (5.6)	3 (5.1)	9 (8.5)

AB, hormonal add-back therapy (estradiol 1 mg/norethindrone acetate 0.5 mg once daily); ELA, elagolix 200 mg twice daily; TEAE, treatment-emergent adverse event.

a Coded using the Medical Dictionary for Regulatory Activities version 26.1.

b Patients are counted once in each row, regardless of the number of events they may have reported.

**Table 4:** All SAEs occurring during the open-label treatment period

SAEs, n (%)^a^^,^^b^	ELA+AB/ELA+AB (n=215)	ELA/ELA+AB (n=59)	Placebo/ELA+AB (n=106)
Lower abdominal pain	0	1 (1.7)	0
Constipation	1 (0.5)	0	0
Food poisoning	0	0	1 (0.9)
Nausea	0	1 (1.7)	0
Cholecystitis	1 (0.5)	0	0
Appendicitis	0	0	1 (0.9)
COVID-19	1 (0.5)	0	0
COVID-19 pneumonia	1 (0.5)	0	0
Gastroenteritis	0	0	1 (0.9)
Alcohol poisoning	1 (0.5)	0	0
Obesity	1 (0.5)	0	0
Flank pain	0	0	1 (0.9)
Migraine	0	0	1 (0.9)
Seizure	1 (0.5)	0	0
Suicidal ideation	0	0	1 (0.9)
Endometriosis	1 (0.5)	0	0
Heavy menstrual bleeding	1 (0.5)	0	0
Ovarian cyst ruptured	0	1 (1.7)	0
Asthma	1 (0.5)	0	0
Abortion induced	1 (0.5)	0	0
Thrombosis	0	0	1 (0.9)

AB, hormonal add-back therapy (estradiol 1 mg/norethindrone acetate 0.5 mg once daily); ELA, elagolix 200 mg twice daily; SAE, serious adverse event.

a Coded using the Medical Dictionary for Regulatory Activities version 26.1.

b Patients are counted once in each row, regardless of the number of events they may have reported.

During the open-label treatment period, 8 patients had pregnancies (ELA+AB/ELA+AB, 7 [3.3%]; ELA/ELA+AB, 0; placebo/ELA+AB, 1 [0.9%]) resulting in 5 live births at term (4 in the ELA+AB/ELA+AB group, 1 in the placebo/ELA+AB group), 1 spontaneous abortion (ELA+AB/ELA+AB group), and 1 termination (ELA+AB/ELA+AB group; Supplemental Table 5). One documented pregnancy (ELA+AB/ELA+AB group) was later attributed to human chorionic gonadotropin injection.

**Figure 2:**
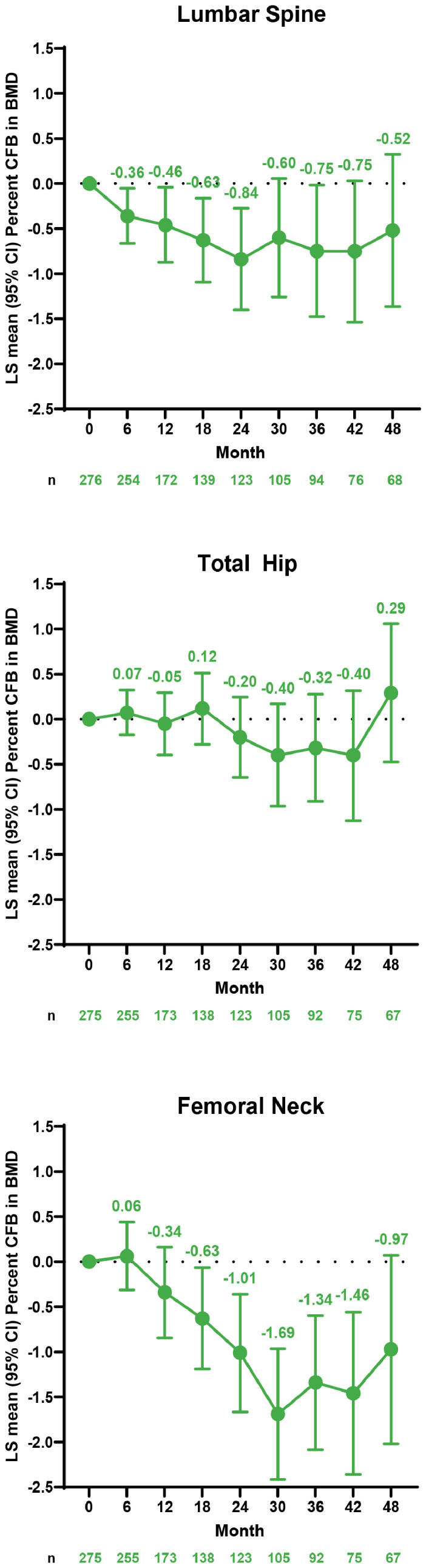
CFB in BMD with up to 48 months of treatment with ELA+AB therapy AB, hormonal add-back therapy (estradiol 1 mg/norethindrone acetate 0.5 mg once daily); BMD, bone mineral density; CFB, change from baseline; ELA, elagolix 200 mg twice daily; LS, least squares.

BMD remained generally stable over the 48-month treatment period. The LS mean percent CFB to month 48 decreased <2% across all anatomic sites in all treatment groups (Table 5). Likewise, patients in the ELA+AB/ELA+AB treatment group had an LS mean percent decrease from baseline <2% at all study time points throughout the placebo-controlled and open-label treatment periods in all anatomic sites examined (Figure 2). Among patients in the ELA+AB/ELA+AB group with at least 48 months of treatment and a BMD decrease at the final on-treatment visit, the majority (lumbar spine, 13 [59.1%]; total hip, 8 [53.3%]; femoral neck, 12 [57.1%]) partially or fully recovered BMD (defined as recovery ≥0%) at 12 months after treatment cessation (Table 6).

**Table 5:** CFB in BMD at month 48

	ELA+AB/ELA+AB (n=67)		ELA/ELA+AB (n=11)		Placebo/ELA+AB (n=29)	
Region	LS mean % CFB (SE)	95% CI	LS mean % CFB (SE)	95% CI	LS mean % CFB (SE)	95% CI
Lumbar spine	−0.52 (0.43)^a^	(−1.37, 0.32)	−0.47 (1.07)	(−2.58, 1.65)	−1.74 (0.66)^b^	(−3.05, −0.43)
Total hip	0.29 (0.39)	(−0.48, 1.06)	−0.13 (0.97)	(−2.05, 1.80)	−1.08 (0.59)	(−2.24, 0.09)
Femoral neck	−0.97 (0.53)	(−2.02, 0.07)	−1.72 (1.31)	(−4.33, 0.88)	−1.53 (0.80)	(−3.12, 0.06)

AB, hormonal add-back therapy (estradiol 1 mg/norethindrone acetate 0.5 mg once daily); BMD, bone mineral density; CFB, change from baseline; ELA, elagolix 200 mg twice daily.

a n=68.

b n=28.

Kim. Safety of 48 months of elagolix with add-back therapy. Am J Obstet Gynecol 2025.

**Table 6:** Categorical percent recovery^a^ from final on-treatment BMD measurement at 12 months PTFU visit in the ELA+AB/ELA+AB group^b^

Recovery, %	Lumbar Spine (n=22) n (%)	Total Hip (n=15) n (%)	Femoral Neck (n=21) n (%)
<0	9 (40.9)	7 (46.7)	9 (42.9)
≥0 and ≤25	3 (13.6)	2 (13.3)	4 (19.0)
>25 and ≤50	1 (4.5)	1 (6.7)	3 (14.3)
>50 and ≤75	1 (4.5)	0	0
>75 and ≤100	0	1 (6.7)	5 (23.8)
>100	8 (36.4)	4 (26.7)	0

AB, hormonal add-back therapy (estradiol 1 mg/norethindrone acetate 0.5 mg once daily); BMD, bone mineral density; CFB, change from baseline; ELA, elagolix 200 mg twice daily; PTFU, posttreatment follow-up.

a Recovery defined as (%CFB at final on-treatment visit − %CFB at each PTFU visit) / (%CFB at final on-treatment visit) in patients who received treatment through month 48 and had a decrease in BMD at their final on-treatment visit.

b Among patients with at least 48 months of treatment.

## Comment

### Principal findings

In this long-term analysis in premenopausal women with moderate-to-severe endometriosis-associated pain, ELA+AB therapy remained generally well tolerated with up to 48 months of treatment. Most TEAEs reported during the open-label treatment period were nonserious, mild or moderate in severity, and did not result in treatment discontinuation. While bone density decrease was among the most common TEAEs reported (ELA+AB/ELA+AB, 9.8%), relatively few patients discontinued treatment primarily due to T- or Z-score less than −2.5 or BMD decrease >8%. Importantly, the CFB in BMD up to month 48 remained generally stable (<2% decrease) for all treatment groups and for all time points in the ELA+AB/ELA+AB group, indicating that AB therapy can attenuate long-term hypoestrogenic effects of ELA. Among patients in the ELA+AB/ELA+AB group who received at least 48 months of treatment and experienced BMD loss at the final on-treatment visit, the majority (lumbar spine, 59.1%; total hip, 53.3%, and femoral neck, 57.1%) showed at least partial BMD recovery at the 12-month PTFU visit.

### Results in the context of what is known

ELA is the first GnRH agonist approved for endometriosis-associated pain.^13^ Findings from phase 3 studies have demonstrated the efficacy of ELA monotherapy in reducing DYS and NMPP following 6 and 12 months of treatment in women with endometriosis-associated pain.^8^^,^^12^ However, significant decreases in BMD in patients treated with ELA monotherapy have been observed in as few as 6 months of treatment (e.g., lumbar spine, −2.9%),^8^^,^^13^ with greater decreases observed at month 12 (e.g., lumbar spine, −3.9%).^12^ The addition of hormonal AB therapy can attenuate the hypoestrogenic effects of ELA monotherapy on BMD loss (e.g., lumbar spine, −1.1% after 12 months of ELA+AB therapy), while maintaining the efficacy of ELA for DYS and NMPP in women with endometriosis-associated pain.^8^^,^^13^ Likewise, a study of 6 months of treatment with ELA for heavy menstrual bleeding and uterine fibroids demonstrated the benefits of AB therapy on maintaining BMD in patients treated with ELA.^14^ Benefits of AB therapy have also been observed among patients with endometriosis treated with the GnRH agonists leuprolide or relugolix.^15^, ^16^, ^17^

Our long-term safety analysis builds on the previously published 12-month safety data of ELA+AB therapy for moderate-to-severe endometriosis-associated pain.^13^ Similar rates of TEAEs were reported in the 12-month double-blind treatment period and the 36-month open-label treatment period (ELA+AB, 73.8%; ELA+AB/ELA+AB, 74.0%). In addition, the safety profile of ELA+AB therapy in the long-term treatment period reported in our study was consistent with that observed in previous studies.^8^^,^^12^^,^^13^ In most phase 3 trials of both ELA monotherapy and ELA+AB therapy, hot flush (another known hypoestrogenic effect of GnRH agonists) has been among the most commonly reported TEAEs.^8^^,^^12^^,^^13^^,^^18^ Notably, in the long-term open-label treatment period, patients in the ELA+AB/ELA+AB group had lower rates of hot flush TEAEs (4.7%) and SAEs (0.0%) compared with those in the 12-month double-blind treatment period (hot flush, 17.7%; SAEs, 1.0%).^13^

In the 12-month analysis, patients initially randomized to ELA monotherapy demonstrated decreases in BMD at 6 months that were attenuated with the introduction of AB therapy.^13^ Patients in the ELA/ELA+AB group continued to show minimal decrease in BMD from baseline to treatment month 48, as did patients initially randomized to ELA+AB therapy or placebo who received treatment with open-label ELA+AB therapy. These results confirm the long-term effects of AB therapy for mitigating bone loss in patients treated with ELA.

### Clinical implications

Endometriosis is a chronic condition that requires long-term management.^2^ While ELA has demonstrated efficacy for managing moderate-to-severe endometriosis-associated pain, it is only indicated for up to 6 months of treatment as a monotherapy due to concerns over bone loss.^11^ To our knowledge, this study is the longest safety analysis of ELA+AB therapy for endometriosis-associated pain. The results in this analysis demonstrate an enduring safety profile, which includes generally stable BMD, that extends beyond 12 months of continuous treatment with ELA+AB therapy.

Despite the overall stability of BMD observed throughout the treatment periods, some patients did show BMD loss over time, even with the inclusion of AB therapy. Bone density decrease was listed among the most common TEAEs, and not all patients with BMD decreases showed evidence of BMD restoration during the PTFU period. Regular monitoring using DXA is recommended for patients receiving long-term treatment with ELA so that patients experiencing BMD loss are detected early and treatment is adjusted accordingly.

### Research implications

This study confirms the long-term (up to 48 months) safety of treatment with ELA+AB for moderate-to-severe endometriosis-associated pain. However, an unmet need remains for treatment options for endometriosis-associated pain that do not involve surgery or hormonal treatment that can help patients effectively manage symptoms long-term.

### Strengths and limitations

This study represents the longest safety analysis of ELA+AB therapy to date. However, after the 12-month randomized, placebo-controlled treatment period, all patients received open-label ELA+AB therapy with no comparator arm. As an open-label descriptive phase of the trial, no inferential statistics could be performed, and patient-reported outcomes were not measured, which limits our ability to assess long-term efficacy of ELA+AB therapy. A further limitation is that the study population was only located in North America, <20% of the study population were non-White and <20% were Hispanic or Latino, possibly limiting generalizability. Additionally, as this study required patients to discontinue treatment if they experienced substantial decreases in BMD, there may have been selection bias due to necessary discontinuations, particularly for BMD measurements in later periods. Importantly, few patients discontinued the study due primarily to DXA measures, and <11% of patients in any treatment group had BMD decrease as a TEAE. Nevertheless, the discontinuation rate in this study was notable, but not uncommon for long-term studies. Imputation methods were not used to analyze these long-term safety data, as the aim of this analysis was to accurately capture the effects of long-term treatment with ELA+AB. Primary efficacy and safety results for ELA+AB therapy have been reported.^13^

## Conclusions

In premenopausal women, treatment with ELA+AB therapy for endometriosis-associated pain for up to 48 months demonstrated favorable tolerability and a safety profile that was consistent with findings from previous studies.^8^^,^^12^^,^^13^ Importantly, the incidence of hot flush was reduced in this analysis relative to past studies of ELA+AB.^13^ BMD remained generally stable over time, demonstrating the enduring benefits of AB therapy for mitigating the potential hypoestrogenic effects of ELA monotherapy. Results from this analysis support the long-term use of ELA+AB therapy for endometriosis-associated pain.

## Patient consent

The study was conducted in accordance with Good Clinical Practice, the International Council for Harmonization, the Declaration of Helsinki, and all applicable local regulations and guidelines. All relevant study materials (including protocols, informed consent forms, etc) received independent ethics committee/institutional review board (IRB) approval before the conduct of the trial (approval received March 4, 2016; IRB reference number: 201601247). Patients provided written informed consent before participation.

## Clinical trial registration

Date of registration: July 7, 2017

Date of initial participant enrollment: July 7, 2017

Trial identification number: NCT03213457

https://clinicaltrials.gov/ct2/show/NCT03213457

## Data sharing statement

• Will individual participant data be available (including data dictionaries)? Yes, on reasonable request based on qualified research (please see more details in the answer to question “e” below)
• What data in particular will be shared? Individual participant data that underlie the results reported in this article, after deidentification (text, tables, figures, and appendices)
• What other documents will be available (eg, study protocol, statistical analysis plan, etc)? Study Protocol, Statistical Analysis Plan
• When will data be available (start and end dates)? Data requests can be submitted at any time after approval in the United States and Europe and after acceptance of this manuscript for publication. These data will be accessible for 12 months, with possible extensions considered.
• How will data be shared (including with whom, for what types of analyses, and by what mechanism)? AbbVie is committed to responsible data sharing regarding the clinical trials we sponsor. This includes access to anonymized individual and trial-level data (analysis data sets), as well as other information (eg, protocols, clinical study reports, or analysis plans), as long as the trials are not part of an ongoing or planned regulatory submission. This includes requests for clinical trial data for unlicensed products and indications. These clinical trial data can be requested by any qualified researchers who engage in rigorous, independent scientific research and will be provided following review and approval of a research proposal, Statistical Analysis Plan (SAP), and execution of a Data Sharing Agreement (DSA). For more information on the process or to submit a request, visit the following link: https://vivli.org/ourmember/abbvie/ then select “Home.”

## Tweetable statement

“Long-Term Safety of elagolix With Add-Back Therapy in Women With Endometriosis-Associated Pain: A 48-Month Treatment Trial and 12-Month Post-Treatment Follow-Up” is now published.

## CRediT authorship contribution statement

**Jin Hee Kim:** Writing – review & editing, Supervision, Investigation, Data curation. **Charles E. Miller:** Writing – review & editing, Investigation. **James A. Simon:** Writing – review & editing, Supervision, Project administration, Investigation, Funding acquisition. **James W. Thomas:** Writing – review & editing, Methodology, Formal analysis, Conceptualization. **Anna Chan:** Writing – review & editing, Supervision, Project administration, Data curation. **Michael C. Snabes:** Writing – review & editing, Supervision, Methodology, Conceptualization.

## Appendix Supplementary materials

Image, application 1

## Footnotes

Supplementary material associated with this article can be found, in the online version, at doi:10.1016/j.xagr.2025.100584.
