# Supplementary material for: Safety of 48 months of elagolix with add-back therapy for endometriosis-associated pain
Source: AJOG Glob Rep. 2025 Nov 7;6(1):100584. doi: 10.1016/j.xagr.2025.100584 (PMC12769792; doi:10.1016/j.xagr.2025.100584)
Supplement: Supplementary file 1 [file mmc1.pdf]

SUPPLEMENTAL DATA

Long-Term Safety of Elagolix With Add-Back Therapy in Women With Endometriosis-Associated Pain: A 48-Month Treatment Clinical Trial and 12-Month Post-Treatment Follow-Up

Jin Hee Kim, MD[a]; Charles E. Miller, MD[b,c]; James A. Simon, MD[d]; James W. Thomas, MS[e]; Anna Chan, PharmD[e]; Michael C. Snabes, MD, PhD[e]

[a]From the Department of Obstetrics and Gynecology, Columbia University, 51 West 51st Street, Suite 320, New York, NY 10019, USA; [b]Department of Minimally Invasive Gynecologic Surgery, Advocate Lutheran General Hospital, 1775 Dempster Street, Park Ridge, IL 60068, USA; [c]The Advanced Gynecologic Surgery Institute, 120 Pam Davis Drive, Suite 100, Naperville, IL 60540, Unites States; [d]George Washington University School of Medicine, IntimMedicine Specialists, 1850 M St. NW, Suite 450, Washington, DC 20036, USA; [e]AbbVie Inc., 1 N. Waukegan Road, North Chicago, IL 60064, USA.

Supplemental Table 1. Exposure to ELA+AB Therapy During the Open-Label Treatment Period

Drug Exposure	ELA+AB/ELA+AB (n = 215)	ELA/ELA+AB (n = 59)	Placebo/ELA+AB (n = 106)
Duration, days, mean (SD)	642.7 (359.5)	560.1 (371.6)	619.4 (373.3)
Duration interval, days, n (%)			
1 – <84	17 (7.9)	4 (6.8)	9 (8.5)
84 – <168	11 (5.1)	9 (15.3)	7 (6.6)
168 – <252	16 (7.4)	2 (3.4)	6 (5.7)
252 – <336	14 (6.5)	6 (10.2)	14 (13.2)
336 – <420	14 (6.5)	4 (6.8)	4 (3.8)
420 – <504	8 (3.7)	3 (5.1)	6 (5.7)
504 – <588	15 (7.0)	4 (6.8)	3 (2.8)
588 – <672	10 (4.7)	0	5 (4.7)
672 – <756	9 (4.2)	5 (8.5)	4 (3.8)
756 – <840	7 (3.3)	2 (3.4)	3 (2.8)
840 – <924	8 (3.7)	2 (3.4)	2 (1.9)
924 – <1008	22 (10.2)	4 (6.8)	8 (7.5)
≥1008	64 (29.8)	14 (23.7)	35 (33.0)

AB, hormonal add-back therapy (estradiol 1 mg/norethindrone acetate 0.5 mg once daily); ELA, elagolix 200 mg twice daily.

Supplemental Table 2. Exposure-Adjusted Event Rates During the Open-Label Treatment Period

TEAEs,<br>Events (Events/100PY)	ELA+AB/ELA+AB<br>(n = 215)<br>(PY = 395.4)	ELA/ELA+AB<br>(n = 59)<br>(PY = 95.2)	Placebo/ELA+AB<br>(n = 106)<br>(PY = 188.2)
Any TEAE	741 (187.4)	217 (227.9)	344 (182.8)
TEAE possibly related to study drug[a]	154 (38.9)	65 (68.3)	135 (71.7)
Severe TEAE	32 (8.1)	4 (4.2)	12 (6.4)
SAE	11 (2.8)	3 (3.2)	7 (3.7)
TEAE leading to discontinuation	51 (12.9)	11 (11.6)	23 (12.2)
Deaths	0	0	0
Most common[b] TEAEs			
COVID-19	33 (8.3)	8 (8.4)	10 (5.3)
Sinusitis	28 (7.1)	5 (5.3)	13 (6.9)
Bone density decreased	21 (5.3)	6 (6.3)	7 (3.7)
Anxiety	20 (5.1)	3 (3.2)	7 (3.7)
Urinary tract infection	19 (4.8)	7 (7.4)	7 (3.7)
Depression	18 (4.6)	2 (2.1)	3 (1.6)
Vulvovaginal mycotic infection	18 (4.6)	5 (5.3)	4 (2.1)
Back pain	17 (4.3)	3 (3.2)	2 (1.1)

AB, hormonal add-back therapy (estradiol 1 mg/norethindrone acetate 0.5 mg once daily); ELA, elagolix 200 mg twice daily; PY, patient-years; SAE, serious adverse event; TEAE, treatment-emergent adverse event.

[a]As assessed by the investigator.

[b]Occurring at a rate ≥4.0 events/100 PY in the ELA+AB/ELA+AB group.

Supplemental Table 3. Most Common Severe AEs Occurring During the Open-Label Treatment Period

Severe AEs Occurring in ≥2 Patients in Any Treatment Group, n (%)[a,b]	ELA+AB/ELA+AB (n = 215)	ELA/ELA+AB (n = 59)	Placebo/ELA+AB (n = 106)
Depression	3 (1.4)	0	0
Suicidal ideation	2 (0.9)	0	1 (0.9)
COVID-19	2 (0.9)	0	0
Endometriosis	2 (0.9)	0	0
Night sweats	2 (0.9)	0	0

AB, hormonal add-back therapy (estradiol 1 mg/norethindrone acetate 0.5 mg once daily); AE, adverse event; ELA, elagolix 200 mg twice daily.

[a]Coded using the Medical Dictionary for Regulatory Activities version 26.1.

[b]Patients are counted once in each row, regardless of the number of events they may have reported.

Supplemental Table 4. Most Common TEAEs Considered Related to the Study Drug Occurring During the Open-Label Treatment Period

TEAEs Considered Related to the Study Drug Occurring in >2.0% of Patients in Any Group, n (%)[a,b,c]	ELA+AB/ELA+AB (n = 215)	ELA/ELA+AB (n = 59)	Placebo/ELA+AB (n = 106)
Bone density decreased	21 (9.8)	6 (10.2)	7 (6.6)
Depression	10 (4.7)	1 (1.7)	2 (1.9)
Hot flush	9 (4.2)	2 (3.4)	8 (7.5)
Weight increased	5 (2.3)	1 (1.7)	1 (0.9)
Night sweats	5 (2.3)	0	3 (2.8)
Suicidal ideation	4 (1.9)	2 (3.4)	2 (1.9)
Anxiety	3 (1.4)	2 (3.4)	5 (4.7)
Headache	3 (1.4)	1 (1.7)	5 (4.7)
Migraine	2 (0.9)	2 (3.4)	6 (5.7)
Nausea	2 (0.9)	1 (1.7)	7 (6.6)
Insomnia	2 (0.9)	0	5 (4.7)
Vulvovaginal mycotic infection	1 (0.5)	3 (5.1)	0
Fatigue	1 (0.5)	2 (3.4)	1 (0.9)
Osteopenia	0	2 (3.4)	1 (0.9)

AB, hormonal add-back therapy (estradiol 1 mg/norethindrone acetate 0.5 mg once daily); ELA, elagolix 200 mg twice daily; TEAE, treatment-emergent adverse event.

[a]Coded using the Medical Dictionary for Regulatory Activities version 26.1.

[b]Patients are counted once in each row, regardless of the number of events they may have reported.

[c]Relationship with study drug was assessed by the investigator.

Supplemental Table 5. Summary of On-Treatment Pregnancy Outcomes During the Open-Label Treatment Period

Outcome, n	ELA+AB/ELA+AB (n = 215)	ELA/ELA+AB (n = 59)	Placebo/ELA+AB (n = 106)
Pregnancies	7[a]	0	1
Live births	4	0	1
Births at term (37 to <42 weeks)	4	0	1
Congenital anomaly	0	0	0
Still birth	0	0	0
Spontaneous abortion	1	0	0
Pregnancy termination	1	0	0
Ectopic pregnancy	0	0	0

AB, hormonal add-back therapy (estradiol 1 mg/norethindrone acetate 0.5 mg once daily); ELA, elagolix 200 mg twice daily; hCG, human chorionic gonadotropin.

[a]One participant had a positive hCG test result following an injection of hCG, but was not pregnant.
